# Relationship between HLA-DQ Gene Polymorphism and Hepatitis B Virus Infection

**DOI:** 10.1155/2017/9679843

**Published:** 2017-04-23

**Authors:** Tao Xu, Meiqun Sun, Hongtao Wang

**Affiliations:** ^1^Department of Clinical Laboratory, Bengbu Medical College, Bengbu, Anhui 233030, China; ^2^Department of Microbiology and Immunology, Medical School of Southeast University, Nanjing, Jiangsu 210009, China; ^3^Department of Histology and Embryology, Bengbu Medical College, Bengbu, Anhui 233030, China; ^4^Department of Immunology, Bengbu Medical College, Bengbu, Anhui 233030, China; ^5^Anhui Key Laboratory of Infection and Immunity, Bengbu Medical College, Bengbu, Anhui 233030, China

## Abstract

Hepatitis B virus (HBV) infection is the predominant risk factor for chronic hepatitis B (CHB). The association between HBV infection and human leukocyte antigen- (HLA-) DQ polymorphism (rs2856718 and rs7453920) has been demonstrated in other studies; however, the results were controversial or inconclusive. Therefore, to derive a more precise estimation of the association, a meta-analysis was performed. Crude odds ratios (ORs) and their 95% confidence intervals (CIs) were used to assess the strength of association between HLA-DQ polymorphism (rs2856718 and rs7453920) and HBV infection risk. A total of 11 articles were used to evaluate the effect of the two polymorphisms on risk of HBV infection. The pooled data showed that HLA-DQ rs2856718-G polymorphism showed protection against HBV infection, and rs2856718-A was a risk factor for chronic HBV infection. The pooled risk estimates indicated that HLA-DQ rs7453920-A polymorphism was associated with decreased risk of HBV infection, and rs7453920-G serves as a risk factor in HBV infection. However, these stratified analyses were lacking credibility due to the limitation of correlational study numbers; further investigation on a large population and different ethnicities is warranted.

## 1. Introduction

Hepatitis B virus (HBV) infection represents a major global health problem, with more than 2 billion people having a history of HBV infection, of whom 400 million are suffering from chronic HBV infection [1, 2]. Recent estimates suggest that HBV infection caused 686,000 deaths in 2013 [3]. In developed countries, the chronic hepatitis B (CHB) infection is relatively rare and acquired primarily in adulthood, whereas in underdeveloped countries, such as Asia and most of Africa, CHB infection is common and usually acquired perinatally or in childhood [1]. Based on the National Disease Supervision Information Management System of China, the mean reported incidence of hepatitis B was 84.3 per 100,000 in China between 2005 and 2010 [4]. Most HBV infections which occur in adults are often self-limited, with spontaneous clearance of HBV from the blood and liver and very small proportion of patients with persistent HBV infection (though less than 5%). As for the reason, why some adults could achieve spontaneous clearance and some would develop into HBV infection is not well clarified [5].

Human leukocyte antigen (HLA) genes are located in chromosome 6p21.31, playing a key role in the immune response against HBV infection [6]. Genetic predisposition of HLA class II antigens may contribute to immune imbalance upon HBV infection, leading to chronic inflammation in the liver [7]. HLA-DQ belongs to HLA class II molecules, which are expressed as cell-surface glycoproteins that bind to exogenous antigens and present them to CD4^+^ T cells [8]. HLA-DQ molecules function as a heterodimer of alpha and beta subunit. Those are encoded by the HLA-DQA1 and HLA-DQB1 genes, respectively. HLA-DQs are highly polymorphic especially in exon 2 which encode antigen-binding sites. Therefore, a number of alleles have been declared to be associated with persistent HBV infection [9].

The single-nucleotide polymorphisms (SNPs) rs2856718, located in the intergenic region between HLA-DQA2 and HLA-DQB1, and rs7453920 are located in the first intron of HLA-DQB2 [6]. A transcriptome study showed that the A allele of rs7453920 was associated with higher HLA-DQ mRNA levels in circulating monocytes, which are critical for mounting immune responses [10]. However, no related evidence indicates that polymorphism of HLA-DQB1 (rs2856718) connected with its mRNA expression. Some recent Genome-Wide Association Studies (GWAS) revealed that the SNP in the HLA-DQ region (rs2856718 and rs7453920) was associated with chronic hepatitis B (CHB) infection [9, 11]. Similarly, several other studies conducted on different populations have investigated the role of HLA-DQ gene polymorphism on development of persistent CHB infection [7–9, 11–18]. However, the findings failed to reach a consensus. Furthermore, a single-center study may have an inadequate sample size and lack of statistical power to obtain reliable conclusions. Therefore, we performed a comprehensive meta-analysis to derive a more precise estimation of the relationship between HLA-DQ (rs2856718 and rs7453920) polymorphism and HBV infection risk.

## 2. Materials and Methods

Literature search strategy using a systematic search was conducted by two investigators, independently. All articles were retrieved from PubMed, EMBASE, and CNKI with the latest search update on March 20, 2017. There were not any limitations on language and publication year. The following terms were used: “HLA-DQ”, “chronic HBV infection” or “chronic hepatitis B” or “Hepatitis B Virus” or “HBV clearance”, “polymorphism” or “SNP”, “rs2856718” or “rs7453920”. The references of all retrieved articles and recent reviews were also manually searched for further relevant studies.

### 2.1. Inclusion and Exclusion Criteria

Criteria for eligible studies were as follows: (a) studies evaluating the association between HLA-DQ polymorphism (rs2856718 or rs7453920) and HBV infection; (b) case-control studies; (c) studies with detailed genotype data that can be acquired to calculate the odds ratios (ORs) and 95% confidence intervals (CIs); (d) studies where genotype distribution of control group must be consistent with Hardy-Weinberg equilibrium (HWE); (e) studies published in English or Chinese. Exclusion criteria were as follows: (a) letters, reviews, and case reports; (b) lack of genotype frequency data; (c) duplicate publication. In addition, if multiple studies had overlapping data, only those with complete data were included.

### 2.2. Data Extraction

The data of the eligible studies were extracted in duplicate and crosschecked by two investigators, independently. The following information was collected from each study: the first author, published year, country, genotyping methods, genotype numbers of HBV infection, and the control group for the HLA-DQ polymorphism (rs2856718 and rs7453920). Any disagreements were resolved by discussion with a third investigator.

### 2.3. Statistical Methods

The statistical work was performed by using Stata software version 12.0 (Stata Corporation, College Station, TX). The data from each SNP were divided into HBV infection, control, or natural clearance group. The strength of the association between HBV infection and the HLA-DQ polymorphisms (rs2856718 and rs7453920) was calculated by odds ratios (ORs) and 95% confidence intervals (CIs). Heterogeneity among studies was tested using the Chi-square-based* Q*-test and *I*^2^ tests, based on the heterogeneity test. The pooled OR was estimated using the fixed (*P*_*H*_ > 0.05 or *I*^2^ > 50%) or random (*P*_*H*_ < 0.05 or *I*^2^ > 50%) effects model. Sensitivity analysis was performed by sequentially omitting one study at a time to estimate the stability of the results. In addition, publication bias among studies was assessed using Begg's test and Egger's test; *P*_*Z*_ < 0.05 was considered significant.

## 3. Results

### 3.1. Characteristics of the Included Studies

**Figure 1:**
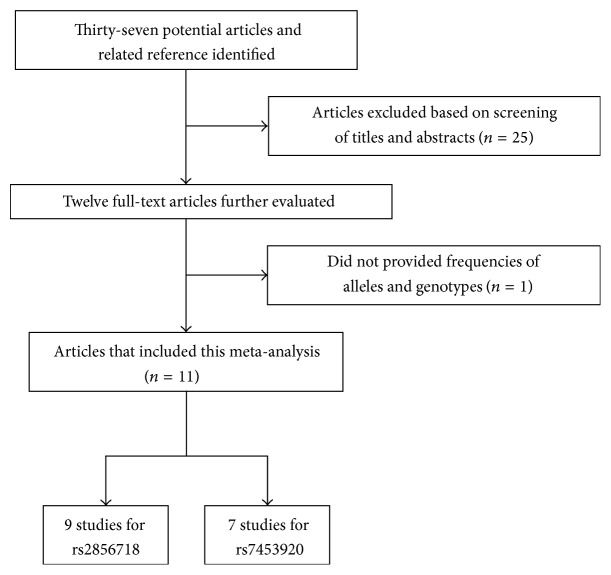
The flow charts of literature search and study selection.

Flow diagrams detailing the selection process of eligible studies are displayed in Figure 1. A total of 37 studies were acquired from PubMed, EMBASE, and China National Knowledge Infrastructure (CNKI). After reviewing the titles, abstracts, and full text, we excluded 26 irrelevant studies. Finally, a total of 11 articles were included in the meta-analysis [7–9, 11–18]. The main characteristics of all eligible studies are shown in Table 1. Furthermore, all of these studies assessed the association between HLA-DQ polymorphisms (rs2856718 or rs7453920) and HBV infection risk. Finally, 9 articles including 12053 HBV carriers, 10043 controls, and 7076 natural clearance were used to assess the risk of HBV infection with HLA-DQ rs2856718 polymorphism. For HLA-DQ rs7453920, 7 articles with 11339 HBV carriers, 7723 controls, and 9851 natural clearance were included.

### 3.2. Association between HLA-DQ rs2856718 and HBV Infection and HBV Natural Clearance

**Figure 2:**
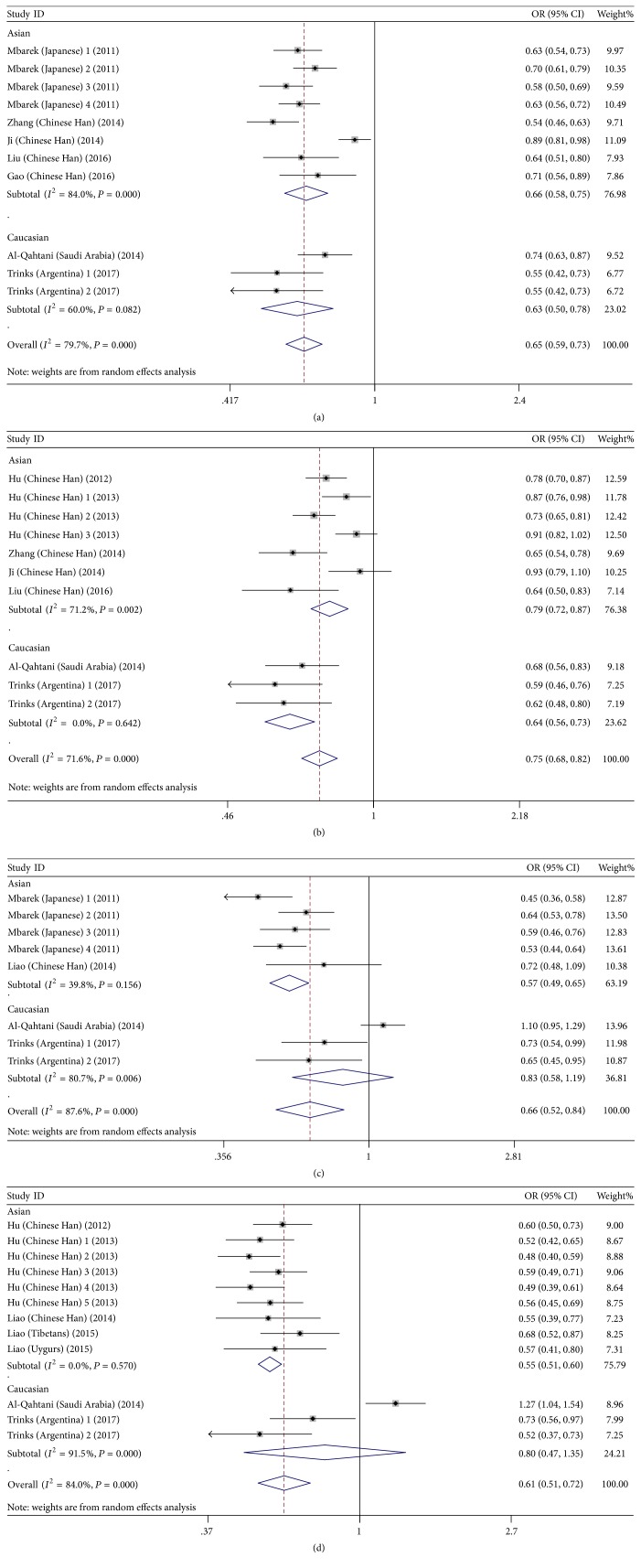
Forest plots for HLA-DQ (rs2856718 and rs7453920) polymorphisms and the risk of HBV infection. (a) Forest plot for rs2856718-G with HBV infection (HBV infection versus control); (b) forest plot for rs2856718-G with HBV infection (HBV infection versus natural clearance); (c) forest plot for rs7453920-A with HBV infection (HBV infection versus control); (d) forest plot for rs7453920-A with HBV infection (HBV infection versus natural clearance).

The results of this meta-analysis are presented in Table 2. The pooled risk estimates indicated that the G alleles of HLA-DQ rs2856718 were associated with decreased risk of HBV infection (AG + GG versus AA: OR = 0.58, 95% CI: (0.52–0.65), *P*_*H*_ = 0.008; GG versus AG + AA: OR = 0.58, 95% CI: (0.47–0.71), *P*_*H*_ < 0.001; GG versus AA: OR = 0.44, 95% CI: 0.36–0.55, *P*_*H*_ < 0.001; AG versus AA: OR = 0.66, 95% CI: 0.62–0.72, *P*_*H*_ = 0.038; G versus A: OR = 0.65, 95% CI: 0.59–0.73, *P*_*H*_ < 0.001), as compared to controls (Figure 2(a)). For HBV clearance, our meta-analysis shown that individuals carrying the HLA-DQ rs2856718-G allele had a significantly lower chance of natural clearance upon HBV infection (AG + GG versus AA: OR = 0.63, 95% CI: 0.52–0.76, *P*_*H*_ < 0.001; GG versus AG + AA: OR = 0.74, 95% CI: 0.63–0.87, *P*_*H*_ = 0.001; GG versus AA: OR = 0.57, 95% CI: 0.48–0.59, *P*_*H*_ = 0.001; AG versus AA: OR = 0.65, 95% CI: 00.51–0.81, *P*_*H*_ < 0.001; G versus A: OR = 0.75, 95% CI: 0.68–0.82, *P*_*H*_ = 0.002) (Figure 2(b)). This suggests that inheriting a single rs2856718-G allele would reduce the risk of an individual to progress into chronic HBV infection.

### 3.3. Association between HLA-DQ rs7453920-A and HBV Infection and HBV Natural Clearance

**Figure 3:**
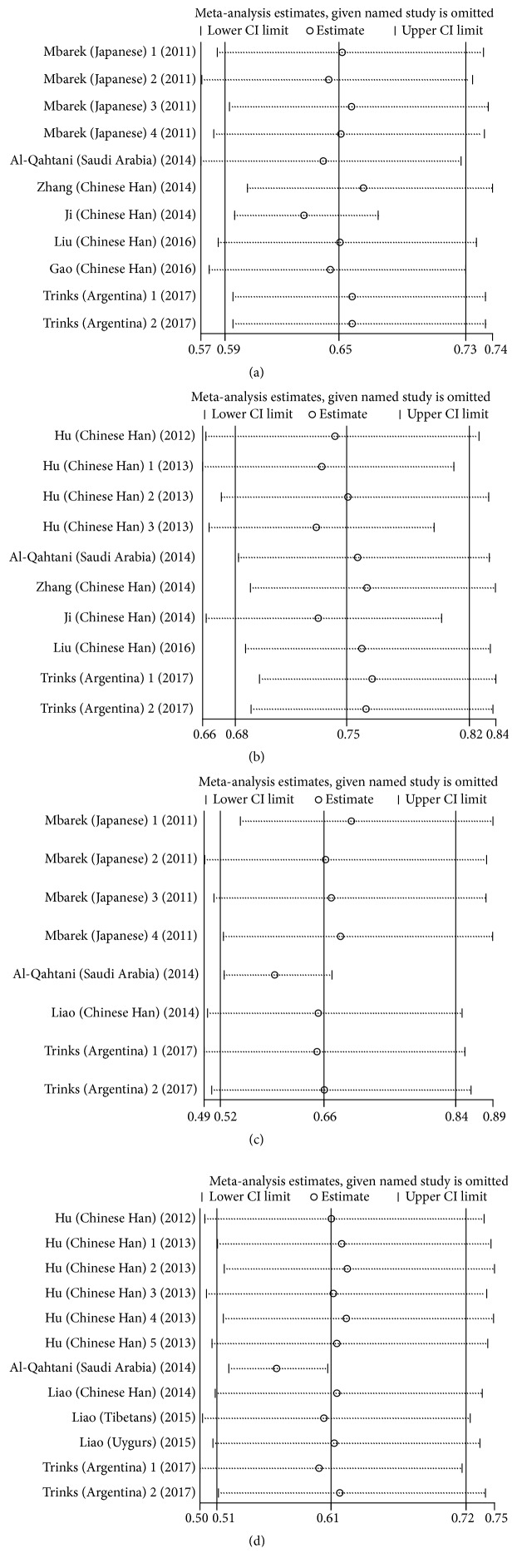
Sensitivity analysis of the pooled ORs and 95% CIs for HLA-DQ (rs2856718 and rs7453920) polymorphisms. (a) The sensitivity analysis results of rs2856718-G with HBV infection (HBV infection versus control); (b) the sensitivity analysis results of rs2856718-G with HBV infection (HBV infection versus natural clearance); (c) the sensitivity analysis results of rs7453920-A with HBV infection (HBV infection versus control); (d) forest plot for rs7453920-A with HBV infection (HBV infection versus natural clearance).

The pooled risk estimates indicated that HLA-DQ rs7453920 allele A was associated with decreased risk of HBV infection (AA + AG versus GG: OR = 0.62, 95% CI: 0.50–0.78, *P*_*H*_ < 0.001; AA versus AG + GG: OR = 0.57, 95% CI: 0.35–0.92, *P*_*H*_ < 0.001; AA versus GG: OR = 0.50, 95% CI: 0.29–0.85, *P*_*H*_ < 0.001; AG versus GG: OR = 0.63, 95% CI: 0.52–0.77, *P*_*H*_ = 0.001; A versus G: OR = 0.66, 95% CI: 0.52–0.84, *P*_*H*_ < 0.001), as compared to healthy controls (Figure 3(a)). For HBV clearance, our meta-analysis has shown that individuals carrying the HLA-DQ rs7453920-A allele had a significantly lower chance of natural clearance upon HBV infection (AA + AG versus GG: OR = 0.57, 95% CI: 0.50–0.66, *P*_*H*_ < 0.001; AA versus AG + GG: OR = 0.54, 95% CI: 0.34–0.86, *P*_*H*_ < 0.001; AA versus GG: OR = 0.48, 95% CI: 0.29–0.78, *P*_*H*_ < 0.001; AG versus GG: OR = 0.58, 95% CI: 0.51–0.65, *P*_*H*_ = 0.006; A versus G: OR = 0.61, 95% CI: 0.51–0.72, *P*_*H*_ < 0.001) (Figure 3(b)). However, allele frequency of rs7453920-A was found more in chronically infected patients when compared to clearance group infection in Saudi Arabian population (Figure 3(b)).

### 3.4. Sensitivity Analysis

The sensitivity analysis was performed to assess the influence of an individual study on the overall OR, and the corresponding pooled ORs were not materially altered (Figure 3).

### 3.5. Publication Bias

**Figure 4:**
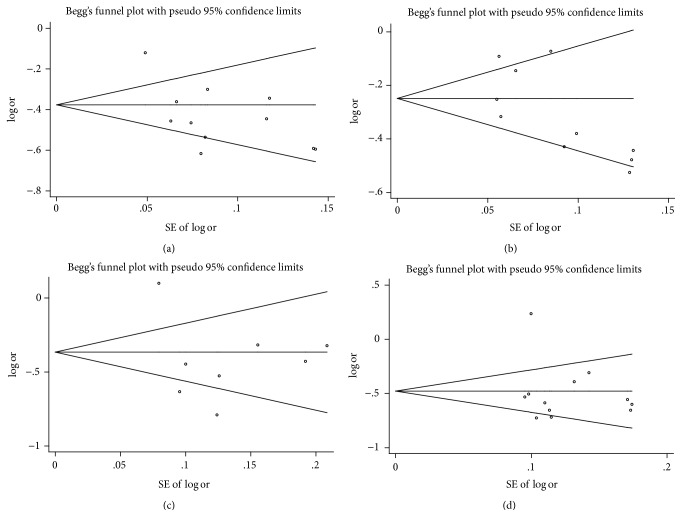
Funnel plot analysis of the association of HLA-DQ (rs2856718 and rs7453920) polymorphisms with HBV infection risk. (a) Funnel plot for rs2856718-G with HBV infection (HBV infection versus control); (b) funnel plot for rs2856718-G with HBV infection (HBV infection versus natural clearance); (c) funnel plot for rs7453920-A with HBV infection (HBV infection versus control); (d) funnel plot for rs7453920-A with HBV infection (HBV infection versus natural clearance).

Publication bias of the included articles was assessed using Begg's funnel plot. The results of funnel plot showed that the shape of the funnel plot seemed symmetrical (Figure 4) and did not show an obvious publication bias for rs2856718 or rs7453920 polymorphism.

## 4. Discussion

A number of epidemiological studies have assessed the associations between HLA-DQ genetic polymorphisms (rs2856718 and rs7453920) and the risk of HBV infection [7–9, 11–18]. For instance, Mbarek et al. have analyzed these loci in three independent Japanese cohorts (2209 CHB cases and 4440 controls) and found significant association of two SNPs (rs2856718 and rs7453920) within the HLA-DQ locus (overall *P* value of 5.98 × 10^−28^ and 3.99 × 10^−37^) [9]. Analyses of Hu et al. showed that HLA-DQ rs2856718 significantly decreased host HCC risk, whereas two SNPs (rs2856718 and rs7453920) were associated with HBV clearance [8, 11]. Zhang et al. confirmed that HLA-DQ rs2856718G is strongly associated with decreased risk of chronic HBV infection and natural clearance [13]. Meanwhile, several other studies conducted on other populations have investigated the role of HLA-DQ polymorphisms (rs2856718 and rs7453920) on the development of persistent chronic HBV infection or its natural clearance [7, 14–18]. However, there were also inconsistent results reported in the previous studies. Al-Qahtani et al. found that the HLA-DQ polymorphism (rs7453920) increased susceptibility to chronic HBV infection was detected in Saudi Arabian patients [12].

To date, numerous studies have been conducted to explore the association of HLA-DQ polymorphisms (rs2856718 and rs7453920) and HBV infection risk, but these findings remain controversial or inconclusive. As is known, the prevalence of HBV infection varies greatly based on region and ethnicity disparity. No previous meta-analysis has comprehensively assessed the associations between the HLA-DQ polymorphisms (rs2856718 and rs7453920) and HBV infection risk. Hence, the current meta-analysis was quite necessary. In order to resolve this conflict, we conducted a meta-analysis on the association between two HLA-DQ polymorphisms (rs2856718 and rs7453920) and HBV infection. Our results showed that SNP rs2856718 was significantly associated with decreased HBV infection risk as well as decreased natural clearance. We investigated the associations between SNP rs7453920 and risk of HBV infection and HBV natural clearance in Asian populations. However, we found that genotype A of HLA-DQ polymorphism (rs7453920) was not associated with the risk of CHB which supported the study of Al-Qahtani et al. [12].

A large number of studies have confirmed that SNPs in HBV-related genes can contribute to individual susceptibility to HBV infection by affecting gene expression and function [19, 20]. For instance, one expression study showed that G alleles of HLA-DP rs3077 and rs9277535 were associated with decreased levels of messenger RNA expression of HLA-DPA1 and HLA-DPB1, respectively, in normal liver tissues [21]. SNP rs3077 was also found to be associated with the methylation status of HLA-DPA1 and HLA-DPB1 in adult cerebellum samples [22]. Therefore, considering that the antigen presentation on the HLA-DQ molecules might be critical for virus elimination and play an important role in the development of HBV infection, we speculated that HLA-DQ polymorphisms (rs2856718 and rs7453920) conferred individual risk for HBV infection by increasing HLA-DQ expression or enhancing HLA-DQ activity.

To the best of our knowledge, this was the first meta-analysis providing comprehensive insights into the effects of rs2856718 and rs7453920 on the risk of HBV infection. Some limitations of this meta-analysis should be recognized. Firstly, we did not have original data for all studies to adjust estimates and perform a more precise analysis, for example, age, gender, body mass index, lifestyle. Secondly, our analysis did not consider the possibility of linkage disequilibrium between gene polymorphisms and the possibility of gene-environment interactions. Thirdly, lack of data for analysis was a constraint for subgroup analysis. Therefore, more studies are needed to get more reliable results.

In conclusion, our meta-analysis suggests that HLA-DQ rs2856718-G is beneficial against HBV infection and rs2856718-A serves as a risk factor in HBV infection. The HLA-DQ rs7453920-A allele was a protective factor for chronic HBV infection, and rs7453920-G serves as a risk factor in HBV infection. However, more large-scale studies are warranted to support our findings.

## Figures and Tables

**Table 1:** Characteristics of the studies included in the meta-analysis.

First author	Year	Country	Ethnicity	Genotyping method	HBV infection	Control	Natural clearance	Polymorphism
					AA/AG/GG	AA/AG/GG	AA/AG/GG	
Mbarek [9]	2011	Japan	Japanese	GWAS	158/226/73	477/1001/568	—	rs2856718
					4/72/382	67/582/1047	—	rs7453920
			First replication	GWAS/invader assay	209/266/127	484/966/572	—	rs2856718
					5/127/471	50/575/1397	—	rs7453920
			Second replication	TaqMan/GWAS/invader assay	128/191/62	325/746/468	—	rs2856718
					4/75/302	53/422/1064	—	rs7453920
			Third replication	Invader assay	465/530/227	216/420/243	—	rs2856718
					14/198/1011	19/245/615	—	rs7453920

Hu [8]	2012	China	Chinese Han	TaqMan	420/674/240	—	344/660/331	rs2856718,
					5/176/1159	—	16/262/1058	rs7453920

Hu [11]	2013	China	Chinese Han	GWAS	302/481/168	—	273/456/208	rs2856718
					4/128/818	—	16/201/685	rs7453920
			Replication Ia	TaqMan	413/606/222	—	305/616/309	rs2856718
					7/139/1074	—	15/271/954	rs7453920
			Replication Ib	TaqMan	305/493/191	—	518/892/392	rs2856718
					8/148/838	—	41/397/1365	rs7453920
			Replication IIa	TaqMan	8/96/876	—	25/256/1103	rs7453920
			Replication IIb	TaqMan	5/126/842	—	18/236/888	rs7453920

Al-Qahtani [12]	2014	Saudi Arabia	Caucasian	DNA sequencing/TaqMan	268/205/204	127/223/155	45/177/73	rs2856718
					144/341/283	90/269/223	34/145/122	rs7453920

Zhang [13]	2014	China	Chinese Han	Flight mass spectrometry	338/345/109	163/225/169	101/177/72	rs2856718

Ji [7]	2014	China	Chinese Han	Real-time PCR	834/1137/422	395/686/245	101/177/72	rs2856718

Liao [14]	2014	China	Chinese Han	High resolution melting	6/48/391	5/33/199	2/87/32	s7453920

Liao [15]	2015	China	Tibetans	High resolution melting	7/96/310	—	13/154/319	rs7453920

Liao [15]	2015	China	Uygurs	High resolution melting	4/58/128	—	17/92/126	rs7453920

Liu [16]	2016	China	Chinese Han	Flight mass spectrometry	170/172/54	81/112/61	50/89/36	rs2856718

Gao [17]	2016	China	Chinese Han	Flight mass spectrometry	88/96/33	163/225/119	—	rs2856718

Trinks [18]	2017	Argentina (central)	Caucasian	TaqMan	62/94/45	31/102/74	52/158/108	rs2856718
					18/67/116	24/85/98	34/135/149	rs7453920

Trinks [18]	2017	Argentina (northwestern)	Caucasian	TaqMan	73/93/34	42/97/62	79/142/92	rs2856718
					7/41/52	10/59/132	22/103/188	rs7453920

—: No data.

**Table 2:** Main results of the meta-analysis of the association between HLA-DQ (rs2856718 and rs7453920) polymorphism and the risk of HBV infection.

Polymorphism	Allele model		Homozygous model		Heterozygous model		Dominant model		Recessive mode	
rs2856718	G versus A		GG versus AA		AG versus AA		(AG + GG) versus AA		GG versus (AG + AA)	
	OR (95% CI)	*P*_*H*_	OR (95% CI)	*P*_*H*_	OR (95% CI)	*P*_*H*_	OR (95% CI)	*P*_*H*_	OR (95% CI)	*P*_*H*_

HBV infection versus control										
Overall	0.65 (0.59–0.73)	<0.001	0.44 (0.36–0.55)	<0.001	0.66 (0.62–0.72)	0.038	0.58 (0.52–0.65)	0.008	0.58 (0.47–0.71)	<0.001
Asian	0.66 (0.58–0.75)	<0.001	0.45 (0.35–0.58)	<0.001	0.70 (0.64–0.76)	0.449	0.61 (0.54–0.69)	0.016	0.56 (0.44–0.71)	<0.001
Caucasian	0.63 (0.50–0.78)	0.0082	0.41 (0.24–0.70)	0.0023	0.46 (0.37–0.58)	0.704	0.48 (0.39–0.59)	0.631	0.63 (0.38–1.06)	0.004
HBV infection versus NC										
Overall	0.75 (0.68–0.82)	<0.001	0.57 (0.48–0.59)	0.001	0.65 (0.51–0.81)	<0.001	0.63 (0.52–0.76)	<0.001	0.74 (0.63–0.87)	<0.001
Asian	0.79 (0.72–0.87)	0.002	0.64 (0.53–0.77)	0.005	0.78 (0.69–0.90)	0.024	0.73 (0.64–0.85)	0.006	0.74 (0.64–0.85)	0.045
Caucasian	0.64 (0.56–0.73)	0.642	0.41 (0.31–0.54)	0.671	0.41 (0.18–0.90)	<0.001	0.41 (0.26–0.65)	0.015	0.72 (0.38–1.38)	<0.001

rs7453920	A versus G		AA versus GG		AG versus GG		AA + AG versus GG		AA versus AG + GG	
	OR (95% CI)	*P*_*H*_	OR (95% CI)	*P*_*H*_	OR (95% CI)	*P*_*H*_	OR (95% CI)	*P*_*H*_	OR (95% CI)	*P*_*H*_

HBV infection versus control										
Overall	0.66 (0.52–0.84)	<0.001	0.50 (0.29–0.85)	<0.001	0.63 (0.52–0.77)	0.001	0.62 (0.50–0.78)	0.111	0.57 (0.35–0.92)	0.001
Asian	0.57 (0.49–0.65)	0.156	0.35 (0.23–0.53)	0.617	0.57 (0.48–0.67)	0.107	0.55 (0.46–0.64)	0.020	0.40 (0.26–0.60)	0.659
Caucasian	0.83 (0.58–1.19)	0.006	0.88 (0.51–1.52)	0.098	0.77 (0.55–1.08)	0.074	0.78 (0.52–1.15)	<0.001	1.01 (0.68–1.51)	0.218
HBV infection versus NC										
Overall	0.61 (0.51–0.72)	<0.001	0.48 (0.29–0.78)	<0.001	0.58 (0.51–0.65)	0.006	0.57 (0.50–0.66)	<0.001	0.54 (0.34–0.86)	<0.001
Asian	0.55 (0.51–0.60)	0.570	0.36 (0.26–0.50)	0.419	0.55 (0.50–0.60)	0.323	0.53 (0.49–0.58)	0.521	0.41 (0.29–0.57)	0.401
Caucasian	0.80 (0.67–1.35)	<0.001	0.83 (0.33–2.08)	0.002	0.70 (0.45–1.08)	0.012	0.72 (0.42–1.24)	<0.001	0.96 (0.44–2.07)	0.007

NC: natural clearance; *P*_*H*_: *P* value of heterogeneity test.
